# Effects of Equal Channel Angular Pressing on the Microstructure and Mechanical Properties of Explosion-Welded Al-Cu Bimetallic Plates

**DOI:** 10.3390/ma18225080

**Published:** 2025-11-08

**Authors:** Krzysztof Żaba, Kinga Ortyl, Ondřej Hilšer, Martin Pastrnak, Łukasz Kuczek, Ilona Różycka, Paweł Pałka, Aleksander Gałka, Tomasz Trzepieciński

**Affiliations:** 1 Department of Metal Working and Physical Metallurgy of Non-Ferrous Metals, Faculty of Non-Ferrous Metals, AGH University of Krakow, al. Adama Mickiewicza 30, 30-059 Cracow, Poland; ortyl@student.agh.edu.pl (K.O.); lukasz.kuczek@agh.edu.pl (Ł.K.); 2 Faculty of Mechanical Engineering, VSB-Technical University of Ostrava, 17. listopadu 2172/15, 708 00 Ostrava-Poruba, Czech Republic; martin.pastrnak@vsb.cz; 3 Department of Materials Science and Engineering of Non-Ferrous Metals, Faculty of Non-Ferrous Metals, AGH University of Krakow, al. Adama Mickiewicza 30, 30-059 Cracow, Poland; rozycka@agh.edu.pl (I.R.); pawel.palka@agh.edu.pl (P.P.); 4 Explomet Gałka, Szulc Spółka Komandytowa, Oświęcimska 100, 46-020 Opole, Poland; aleksander.galka@explomet.pl; 5 Department of Manufacturing Processes and Production Engineering, Faculty of Mechanical Engineering and Aeronautics, Rzeszów University of Technology, al. Powst. Warszawy 8, 35-959 Rzeszów, Poland; tomtrz@prz.edu.pl

**Keywords:** Al/Cu bimetallic plates, ECAP, hardness, mechanical properties, microstructure, plastic working

## Abstract

Explosive welding technology is crucial for the production of large-area plates composed of materials with varying plastic and physical properties. Severe plastic deformation processes increase the mechanical strength of the plates by refining grains and increasing dislocation density. The aim of the research presented in this paper was to analyze the effect of Equal Channel Angular Pressing (ECAP) on the mechanical properties and microstructure of an Al/Cu (EN AW-1050/Cu-ETP) bimetallic plate produced by the explosive welding technology. The ECAP process was carried out at room temperature. The ECAP experiments consisted of 1–3 passes using a die with a channel angle of 90°. The ram speed was 40 mm/min. The study also considered various sample cutting orientations (longitudinal, transverse) and various positions of the bimetallic sample in the die entry channel. Rotating the sample by an angle of 180° between consecutive passes was also considered. To achieve the research objective, static tensile tests, Vickers hardness tests at a load of 4.9 N, and microstructural analysis of the samples using scanning electron microscopy and energy dispersive spectroscopy were carried out. It was found that each subsequent pass in the ECAP process led to a gradual, severe change in the morphology of the Al/Cu interfacial transition layer. The orientation of the cutting plane of the samples was shown to have no effect on the hardness of the bimetallic material. Vickers hardness tests preceded by the ECAP process revealed a more uniform hardness distribution compared to the base material. The orientation of the Al/Cu plate layers in the ECAP die channel clearly influenced the character of the hardness distribution.

## 1. Introduction

The development of unconventional technologies for joining metallic materials is an integral part of industrial and scientific progress today [1]. Joining technologies (such as welding, brazing, soldering, riveting, and adhesive bonding) are of paramount importance to the development of the metalworking industry, enabling the production of complex structures from various metal components [2]. Solid-state welding (SSW) is a family of joining processes that form a strong metallurgical bond between materials without melting them, instead relying on pressure and/or heat to cause intermolecular diffusion at temperatures below the melting point [3,4]. SSW reduces the occurrence of defects typical of conventional welding such as crystallization cracks, porosity, and inclusions [5]. Ensuring the structural integrity of welded joints is essential when predicting fatigue life of welds. A Gaussian variational Bayes network-based model developed by Zhang et al. [6] was used to determine the most influential parameters affecting weld fatigue life. The performance of the proposed method was inferior to Bayesian neural network, back-propagation neural network and Gaussian process regression.

SSW methods include ultrasonic welding, friction welding, diffusion welding, forge welding, cold welding, and explosive welding processes. Ultrasonic welding is based on the transfer of high-frequency mechanical vibration energy to the welded surface [7,8,9]. Ultrasonic vibrations generate frictional heat and pressure at the interface of materials, resulting in their joining in the solid state [10]. Friction welding is a process in which a permanent joint is created by heating the joining surface to the plasticizing temperature as a result of friction between the joined surfaces. Diffusion welding can be performed in the solid state or in the liquid phase. Forge welding is one of the simplest and oldest methods of joining metals [11]. It involves heating metal elements to their plasticization temperature (0.5–0.9 T_melt_) and pressing them together by forging [8]. Cold welding, on the other hand, involves joining two elements at ambient temperature and significant pressure (1000 MPa), causing deformation at their contact interface [8,12].

Explosive welding, also known as explosive cladding, is a solid-state metal joining process that utilizes high mechanical energy to create a metallic joint [8]. During the formation of a metallurgical joint, very little or no melting occurs at the interface between the materials [13]. Advantages of explosive welding over other solid-state joining techniques include the ability to join large-area panels in a single joining operation, as well as joining metals with very different melting points and thermal expansion [14,15]. Explosive cladding does not produce heat-affected zones, so it can effectively join even very thin plates and foils, and the joint is characterized by greater strength than the materials being joined [16,17]. Explosive welding is characterized by a number of interrelated and interdependent process variables, the appropriate combination of which determines the degree of deformation and the conditions for weld formation [18]. The production of multi-layered explosively welded metallic materials can be compared to the electroplating process, which produces a finish coating on the substrate surface of another material [19].

Increasing the mechanical strength of metallic composite materials composed of materials with different properties is desired in many industries [20,21]. Grain size is a key microstructural factor influencing the strength and plastic properties of metals. One of the methods for refining the microstructure of metals and their alloys, and thus increasing their strength, is Severe Plastic Deformation (SPD) [22,23]. The main SPD methods include Equal Channel Angular Pressing (ECAP), High Pressure Torsion (HPT), Accumulative Roll Bonding (ARB), Two-Counter Angular Pressing (TCAP), Constrained Groove Pressing (CGP), multi-directional forging, and Dual Rolls Equal Channel Extrusion (DRECE) [24,25,26]. SPD methods enable grain refinement to sizes of 100–1000 nm, and sometimes even below 100 nm. These technologies introduce high dislocation density into the material, ensuring a uniform, fine-grained microstructure.

Al/Cu bimetallic plates combine the low density and low cost of aluminum with the excellent thermal and electrical conductivity of copper, which opens up opportunities for the development of new, innovative concepts [27]. Further applications can be expanded by increasing the mechanical strength of such composites through the SPD process, which refines grains and increases dislocation density in the material. This paper comprehensively examines the properties of explosively welded Al/Cu plates in the ECAP process for the first time, taking into account different sample orientations, different ECAP processing strategies, and different numbers of passes. The aim of this study was to analyze the effect of the ECAP process parameters on the mechanical properties and microstructure of the Al/Cu bimetallic plates in different processing strategies. Vickers hardness tests, static tensile tests, scanning electron microscopy (SEM), and energy dispersive spectroscopy (EDS) were carried out to achieve this goal.

## 2. Materials and Methods

### 2.1. Test Material

The test material for the ECAP investigations was a bimetallic plate produced by the explosive welding method under industrial conditions at Explomet (Opole, Poland). Two 5 mm-thick plates, Cu-ETP copper (base material) and EN AW-1050 aluminum alloy (clad) were explosively welded. The chemical composition of the EN AW-1050 aluminum alloy and Cu-ETP copper is presented in Table 1 and Table 2, respectively.

**Figure 1:**
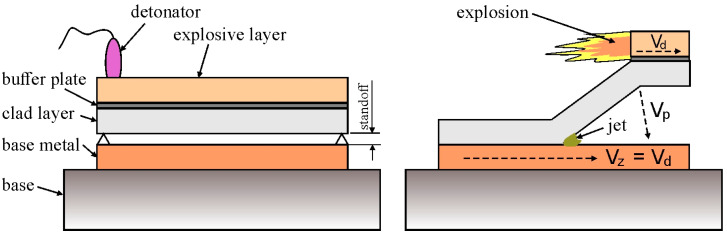
Schematic diagram of explosive welding in a parallel arrangement.

The explosive welding process was carried out in a parallel welding arrangement, in which the plates to be joined are arranged parallel to each other (Figure 1). This system ensures that the welding speed V_z_ is equal to the detonation velocity of the explosive charge V_d_ [16]. The use of a parallel welding arrangement is recommended when the detonation velocity of the explosive V_d_ is lower than the propagation velocity of sound waves in metals V (in metals V_d_ < V). The optimal standoff between the elements should be three times the thickness of the clad layer [8]. The parallel arrangement is suitable for welding large-area and thick plates, ensuring even distribution of the energy from the detonation over the entire welding area.

**Figure 2:**
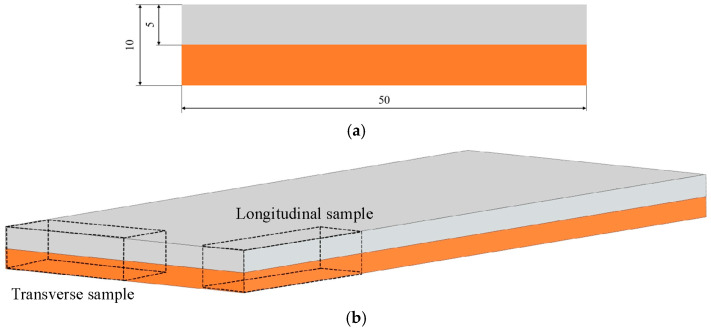
(**a**) dimensions of the test samples and (**b**) simplified diagram of cutting the samples from the base material.

**Figure 3:**
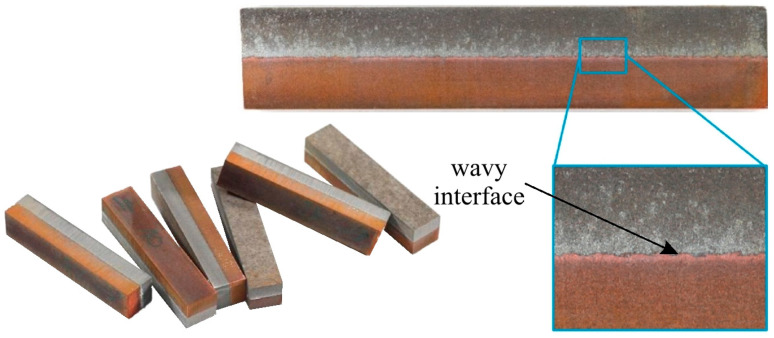
Photograph of sample workpieces cut from the explosion welded bimetallic plate and a magnification of the Al/Cu interfacial transition layer.

An explosion welded bimetallic plate was used as the base material for further testing in the ECAP process. Six samples, 50 mm long and 10 × 10 mm in cross-section, were prepared (Figure 2a). Five of them were cut from the starting material longitudinally to the Al/Cu interfacial transition layer, and the last sample was cut transversely (Figure 2b). Figure 3 presents photographs of selected samples and a magnified view of the Al/Cu interfacial transition layer. A wave-like interfacial transition layer geometry that forms between the two bonded metal plates was observed. This is a typical type of interface in the explosion welding. The wavy morphology of the interface increases the contact area of the base plates and, consequently, the mechanical strength of the weld [30]. The sample cut transversely to the Al/Cu interfacial transition layer was denoted by the digit 5 (ECAP5), while the samples cut longitudinally were denoted by the numbers: 10 (ECAP10), 11 (ECAP11), 12 (ECAP12), 13 (ECAP13) and 14 (ECAP14).

**Figure 4:**
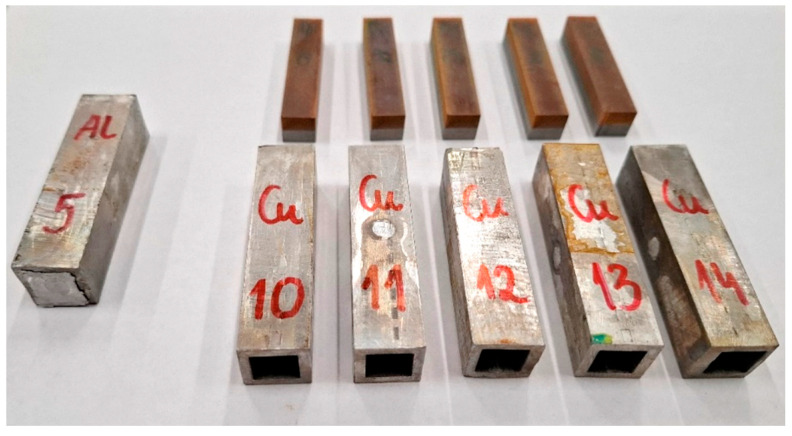
Samples prepared for ECAP testing.

The cut samples were then prepared for ECAP testing by placing them in special annealed EN AW-1360 aluminum alloy shields. The cross-section of the ECAP dies was larger (15 × 15 mm) than the cross-section of the samples (10 × 10 mm), so additional shields were necessary. Figure 4 shows examples of samples before and after preparation for ECAP testing. The samples were tightly placed in the shields to prevent displacement during forming.

### 2.2. Experimental Procedure

#### 2.2.1. Equal Channel Angular Pressing

**Figure 5:**
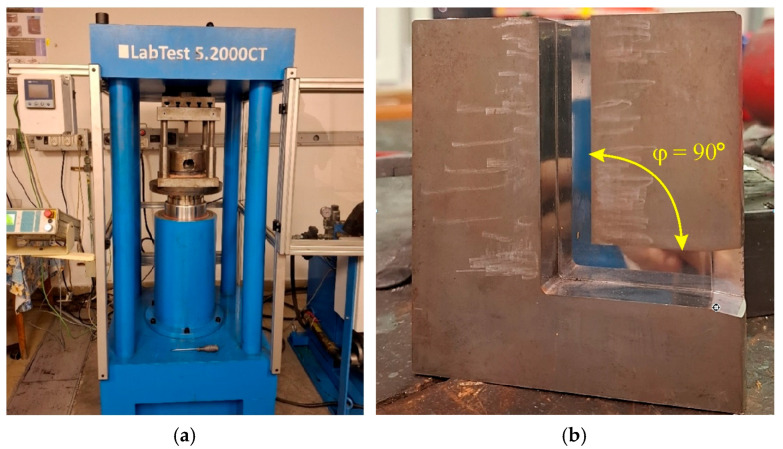
(**a**) Hydraulic press for ECAP testing and (**b**) die view (φ = 90°).

ECAP investigations were conducted at the Faculty of Mechanical Technology at the VŠB—Technical University of Ostrava (Ostrava, Czech Republic). The experimental tests were performed on a LabTest 5.2000CT hydraulic press (LMAT Instruments, São Paulo, Brazil) (Figure 5a) with a maximum ram speed of 400 mm/min and a maximum force of 2 MN.

ECAP tests were performed on two types of samples—cut transversely and longitudinally to the Al/Cu interfacial transition layer. Additionally, the effect of the sample transition layer position within the die entry channel on the material properties of ECAP-processed material was investigated. Increasing the number of material passes through the die channels resulted in a reduction in grain size, which led to increased strain accumulation and increased material strength [31,32,33]. Depending on the sample type, the ECAP processes were performed in up to three passes (Table 3). Between each pass through the die channel, the sample was trimmed and ground before the next pass. Table 3 summarizes all sample designations, along with information on their initial orientation, processing method, and the total number of passes in a given ECAP process.

**Figure 6:**
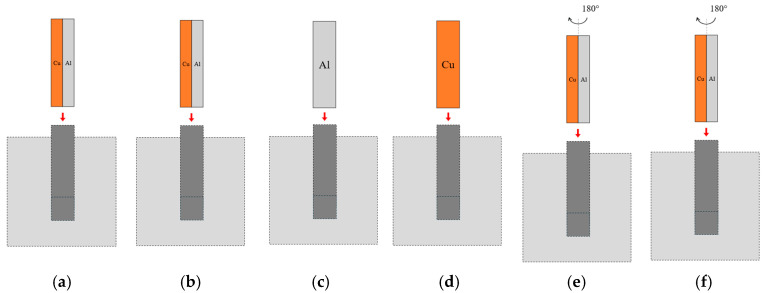
ECAP configurations (front view): (**a**) ECAP5, (**b**) ECAP10, (**c**) ECAP11, (**d**) ECAP12, (**e**) ECAP13, (**f**) ECAP14.

The ECAP process was carried out at cold forming conditions (room temperature). A tool with a channel angle of φ = 90° was selected for testing (Figure 5b). The ram speed was 40 mm/min. Each sample was processed in a different orientation to determine the effect of material arrangement in the die on the mechanical properties of the tested bimetallic material (Table 3). Samples ECAP5 (Figure 6a) and ECAP10 (Figure 6b) were placed in the die entry channel parallel to the outlet channel. Samples ECAP11 (Figure 6c) and ECAP12 (Figure 6d) were placed in the entry channel with the Al and Cu sides facing the die exit hole, respectively. ECAP5, ECAP11, and ECAP12 samples were formed in a single pass. ECAP13 (Figure 6e) and ECAP14 (Figure 6f) samples were placed in the die entry channel parallel to the outlet channel. Forming of ECAP13 and ECAP14 samples was performed in two and three passes, respectively, with 180° rotation between successive passes in a clockwise direction.

#### 2.2.2. Scanning Electron Microscopy

**Figure 7:**
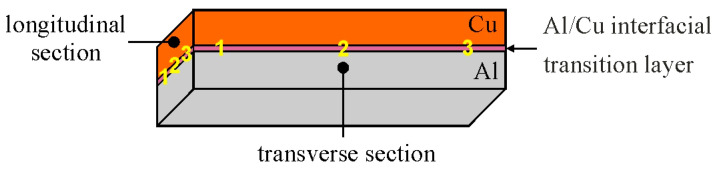
Scheme of microstructural examination of samples—1, 2, 3 in longitudinal section and 1, 2, 3 in transverse section.

Microstructure examination of the base bimetallic material and ECAP-processed samples was performed using a HITACHI SU 70 scanning electron microscope (Hitachi High-Tech, Tokyo, Japan). The microstructure of the base material was observed in three locations on the sample, in two planes: transverse (samples T1, T2, T3) and longitudinal (samples L1, L2, L3) to the Al/Cu interfacial transition layer (Figure 7). All samples were prepared as metallographic microsections, ground on abrasive papers with grit ranging from 200 to 4000 µm and then polished on polishing cloths using DiaDuo diamond suspensions (Struers, Copenhagen, Denmark) with grit ranging from 6 to 3 µm. Final processing involved polishing the microsections in an oxide polishing suspension.

Analysis of chemical composition in the form of elemental mapping and spot EDS examination of selected locations in the ECAP-processed samples were performed.

#### 2.2.3. Hardness

Hardness measurements of the base material and the ECAP-processed samples were carried out using a Wilson^®^ Tukon 2500 Vickers hardness tester (Wilson Instruments, Lake Bluff, IL, USA) at a load of 4.9 N. The indenter was a diamond pyramid with an apex angle of 136°.

**Figure 8:**
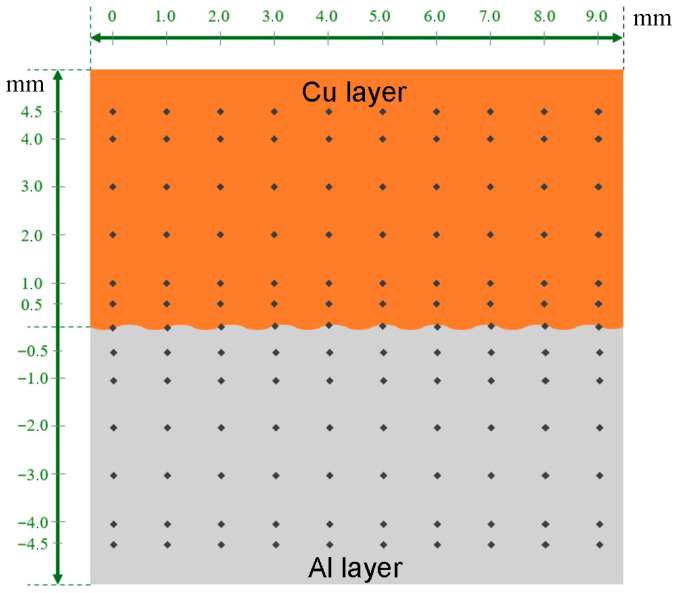
Hardness distribution measurement diagram.

To precisely analyze the hardness of the tested material, a hardness distribution maps were created. For this purpose, measurement indentations were made at the following distances from the Al/Cu interfacial transition layer: 0.5, 1.0, 2.0, 3.0, 4.0, and 4.5 mm in the direction of the Y-axis, and at 1 mm intervals along the X-axis (Figure 8). This resulted in a hardness distribution map across the entire cross-sectional area of the samples. Depending on the sample shape, the number of measurements was reduced and adjusted to the sample dimensions.

#### 2.2.4. Uniaxial Tensile Test

**Figure 9:**
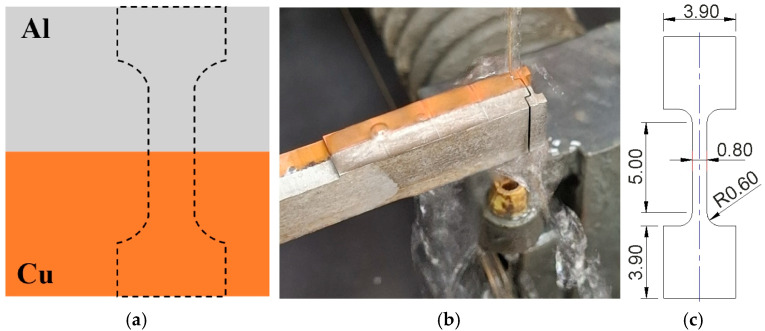
(**a**) Scheme of cutting a sample from a bimetallic plate, (**b**) sample view, and (**c**) sample dimensions.

The static uniaxial tensile test samples were cut transversely to the Al/Cu interfacial transition layer. During testing, the joint interface between the two materials was centered on the gauge length (Figure 9a). The strength tests were carried out on the base material (Figure 9b) and on the ECAP-processed samples. Samples were cut on a wire EDM (Electrical Discharge Machining) machine. Sample dimensions are shown in Figure 9c. The sample thickness was 0.8 mm. The investigations were performed with three repetitions. The static uniaxial tensile test was carried out on a Zwick/Roell Z050 testing machine with a constant strain rate equal to 5 × 10^−3^ s^−1^.

## 3. Results and Discussion

### 3.1. Base Bimetallic Material

#### 3.1.1. Interface Microstructure

**Figure 10:**
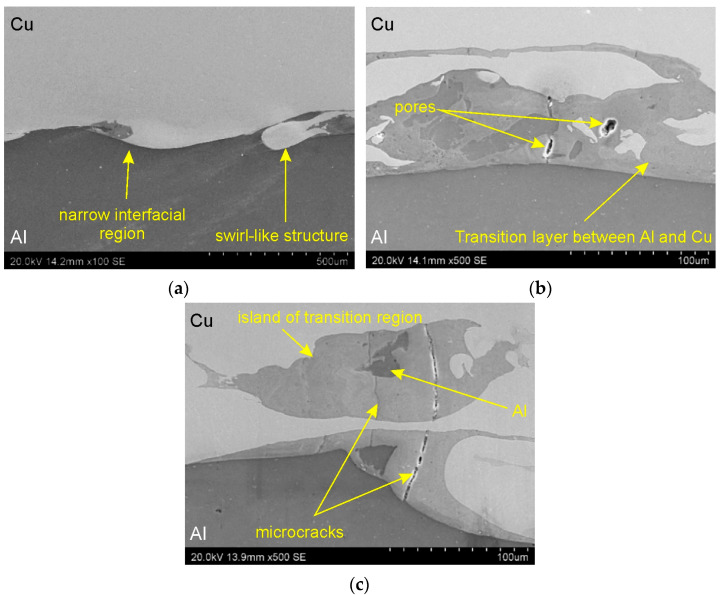
SEM micrographs illustrating the interface layer in cross-section at different magnifications: (**a**) ×100 and (**b**,**c**) ×500.

**Figure 11:**
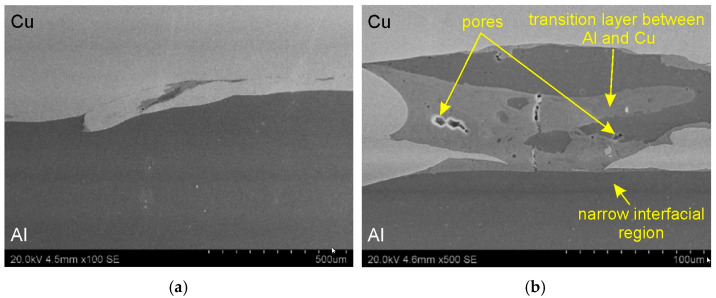
SEM micrographs illustrating the interface layer in longitudinal section at different magnifications: (**a**) ×100 and (**b**) ×500.

Figure 10 and Figure 11 present SEM micrographs illustrating the interface layer in the base bimetallic plate in the transverse and longitudinal sections of the Al/Cu interfacial transition layer. Examination of the micrographs revealed no significant differences resulting from the sample cutting orientation. A comparable microstructure was observed for both sections analyzed. Furthermore, samples cut from three different locations in the material (Figure 7) did not differ in microstructure. The Al/Cu bimetallic plate was characterized by a wave-like geometry that forms between the two bonded metal plates. Wavy interface is consistent with the common theory of explosive welding of Al/Cu material configuration (Al—clad, Cu—base metal). The wave-like geometry interface in explosive welding is a characteristic feature resulting from the high-velocity collision of two plates [34]. This wave formation is initiated by a Kelvin-Helmholtz instability mechanism, where a jetting phenomenon occurs at the collision point [35]. The waves at the weld interface are thought to appear like vortices do when a discontinuity between the flows occurs [36]. According to Bahrani et al. [37], wave formation in explosive welding can be explained by the re-entrant jet which is responsible for the creation of a hump ahead of the collision point. According to Ben-Artzy [38] and Plaksin et al. [39] the formation of the wave is triggered by instabilities arising from the oscillations of the detonation wave transmitted to the phase boundary between the plates.

The presence of swirl-like structures, characteristic of the explosive welding process, was also detected at the interface layer (Figure 10a). At higher magnifications, individual pores (Figure 10b and Figure 11b) and large transition layers (Figure 10b and Figure 11b) between the Al/Cu materials were observed. These layers result from local melting and mixing of the base materials, leading to the formation of intermetallic compounds. Figure 10c shows a characteristic “island” of the transition region that has moved below the surface of the Cu plate. In the central part of this region, a darker area was observed, most likely corresponding to a piece of the aluminum that had been displaced during the explosive welding process.

#### 3.1.2. Vickers Hardness

Vickers hardness measurements were conducted for three longitudinally oriented samples (designated L1–L3), and for three samples oriented transversely to the Al/Cu interfacial transition layer (designated T1–T3). Table 4 summarizes the hardness measurement results for base material samples. The transversely and longitudinally cut samples showed no significant differences in hardness.

**Figure 12:**
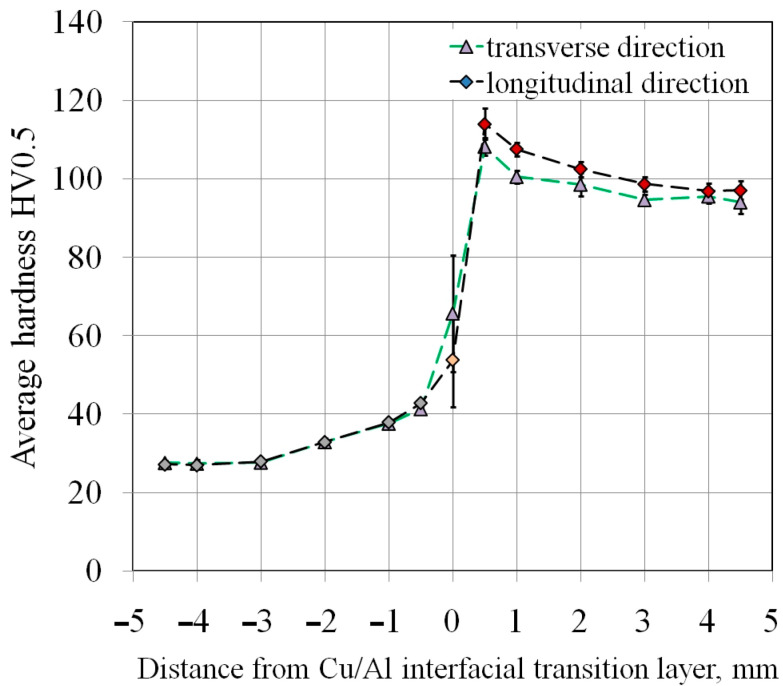
Distribution of material hardness depending on the distance from Al/Cu interfacial transition layer for samples L1 and T1.

The hardness distribution across the sample cross-section indicates a decrease in the hardness of the joined materials with distance from the Al/Cu interfacial transition layer (Figure 12). Large plastic deformation and the presence of alloying compounds at the interface contribute to the increase in hardness of micro-areas [40]. The decrease in hardness with increasing distance from the interface was confirmed in the works of Sun et al. [41] and Kaye et al. [42]. This relationship applies to both analyzed sample orientations. The closer to the outer edge of the materials, the greater the flatness of the graph and the more uniform the measurement results. This conclusion can be generalized to the remaining measurements L2, L3, T2, and T3.

**Figure 13:**
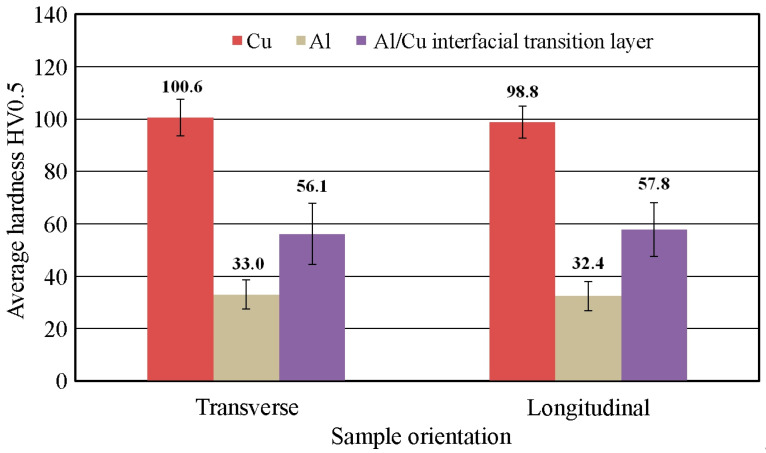
Effect of measurement orientation on average hardness.

Higher hardness of base metal in the Cu and Al layers was observed in the samples cut in longitudinal direction compared to samples cut transversely. The hardness of the Al/Cu interfacial transition layer was higher for the samples cut in the transverse direction of the base metal (Figure 13). However, these differences are very small, and the tests were characterized by large standard deviations of the results. A characteristic relationship was observed based on the hardness analysis of all the base material samples. An uneven hardness distribution was observed within both layers (Al, Cu) of bimetallic plate. The distance from the Al/Cu interfacial transition layer had a significant impact on the hardness. In the close proximity of the Al/Cu transition layer, the highest hardness values were obtained for EN AW-1050 aluminum alloy and Cu-ETP copper. The near-surface areas of the samples, however, were characterized by the lowest hardness values (Figure 12). This material behavior was caused by the bimetallic plate manufacturing process. As a result of the strong collision of the plates during explosive welding, the material in the area of the strongest shock wave impact was subjected to the work hardening phenomenon.

#### 3.1.3. Mechanical Properties

**Figure 14:**
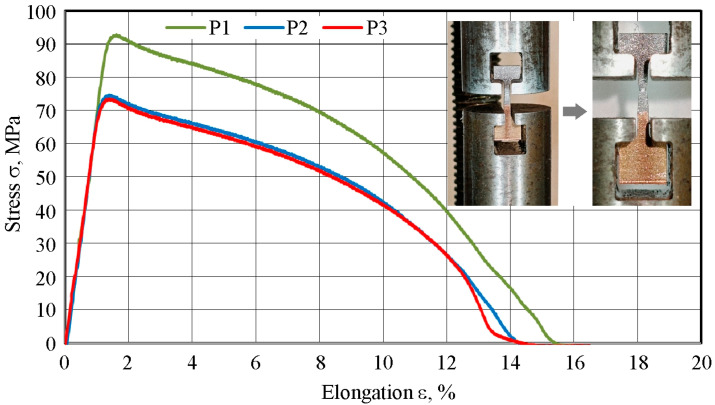
Stress–strain curve of the Al/Cu plate.

Figure 14 presents stress–strain (σ–ε) curves for the samples cut transversely to the Al/Cu interfacial transition layer. Basic mechanical parameter values were determined based on testing three samples (P1, P2, and P3) stretched under identical conditions. The straight initial sections of the curves corresponded to the elastic strains. Based on the slope of these sections, the mean Young’s modulus of the tested bimetallic material was determined (E = 70.1 GPa). The standard deviation value for the modulus of elasticity was 2.4 GPa. This means that the Young’s modulus of the Al/Cu bimetallic plate decreased compared to the clad material (E_Al_ = 60.0 GPa). After exceeding the yield strength, the material underwent permanent deformation, and a further increase in stress led to a rapid achievement of the tensile strength (R_m_). Table 5 presents the averaged results of the basic mechanical properties of the Al/Cu material in the as-received state. The average tensile strength and yield strength were R_m_ = 80.3 MPa and R_p0.2_ = 59.4 MPa, respectively. For comparison, the tensile strength of the EN AW-1050 aluminum alloy is approximately 65–95 MPa, and for Cu-ETP copper it ranges between 200 and 220 MPa. The average total elongation of the Al/Cu material produced by the explosion welding method was A = 14.7%. The samples were fractured in the Al layer zone (Figure 14). The character of the variation of the tensile curves of Al/Cu bimetallic plates is consistent with the results presented by Żaba et al. [43].

### 3.2. Equal Channel Angular Pressed Samples

#### 3.2.1. Mechanical Properties After ECAP

**Figure 15:**
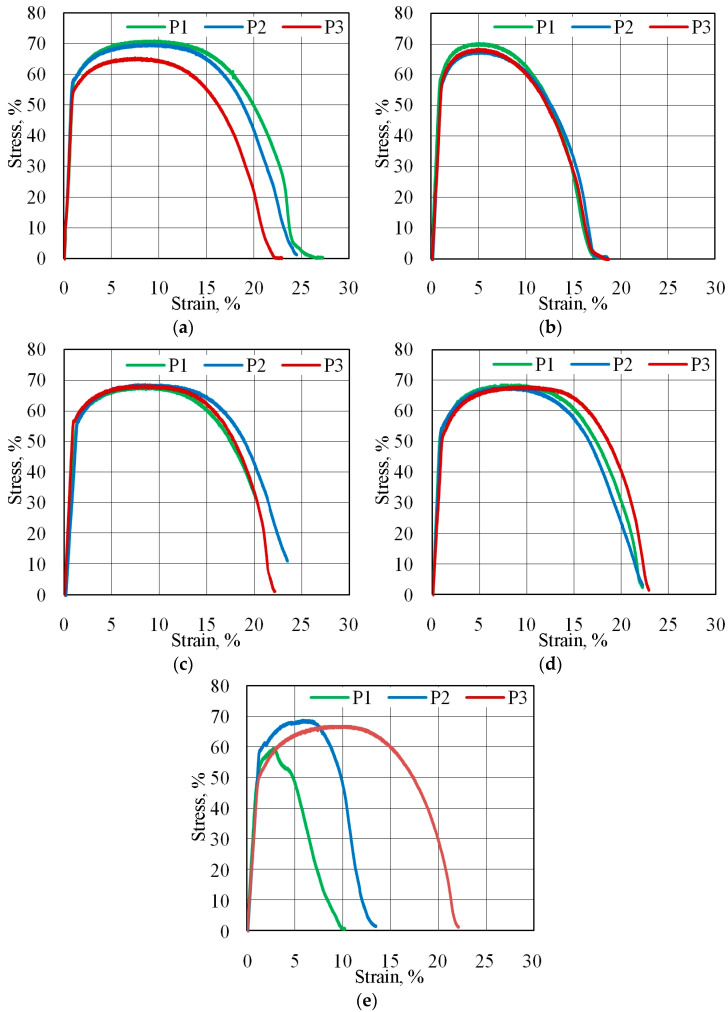
Stress–strain curves obtained from uniaxial tensile tests of ECAPed samples: (**a**) ECAP10, (**b**) ECAP11, (**c**) ECAP12, (**d**) ECAP13 and (**e**) ECAP14.

Figure 15 presents the stress–strain (σ–ε) curves of the ECAP-processed Al/Cu bimetallic plates. All samples were characterized by very similar stress–strain curves. Based on the results obtained for the ECAP5 and ECAP10 samples, no significant differences were found that would indicate the influence of the initial sample orientation on the mechanical properties of the test material. Therefore, this section discusses the results for the samples ECAP10–ECAP14 cut longitudinally to the Al/Cu interface (Table 3). It was noted that after processing the ECAP10 sample (Figure 15a), the tensile strength R_m_ decreased by 15.1 MPa compared to the base material. However, the yield strength of a bimetallic plate increased by 2.3 MPa. Two of the three analyzed ECAP10 samples revealed a physical yield point R_e_. Both variants were characterized by comparable values of tensile strength (R_m_ = 65.4–71.0 MPa) and yield strength (55.5–58.2 MPa). The elongation value varied in the range of 22.5–27.3%. In the case of the ECAP11 sample (Figure 15b), the average tensile strength value decreased by 11.61 MPa compared to the tensile strength of the base metal. A slight decrease (2.56 MPa) in the yield strength was also noted. One of the three analyzed ECAP11 samples revealed a physical yield point. The average elongation decreased by 17.8% compared to the ECAP10 sample. The average tensile strength of the ECAP12 samples (Figure 15c) decreased by 12.01 MPa compared to the base material. A slight decrease (3.01 MPa) in the yield strength was also noted. No significant differences were observed in the measured parameters for the ECAP12 and ECAP13 samples, indicating no direct effect of the sample orientation in the die entry channel on the strength properties of the bimatallic plate. However, all ECAP12 samples exhibited a physical yield point. The tensile strength of the ECAP13 sample (Figure 15d), deformed in two ECAP passes, decreased by 12.32 MPa compared to the tensile strength of the as-received sample. A reduction in the yield strength by 6.07 MPa and the presence of a physical yield point were also observed. The initial segments of the recorded curves P1, P2, and P3 for the ECAP14 sample deformed in three passes were similar; however, significant discrepancies were observed at higher strains (Figure 15e). These differences may result from excessive work hardening as a result of three-pass ECAP processing. Results obtained during tensile tests are in good correlation with literature data. Due to the layered structure of the tested bimetallic samples, defects could develop at the interface and local crack initiation could occur [44]. This suggests that the samples may have a heterogeneous microstructure and, in some cases, the presence of local defects leading to premature fracture. Increasing the number of passes results in a finer structure and a higher fraction of high-angle boundaries, which increases the material’s strength and reduces ductility [45]. Increasing the number of passes with sample rotation during the ECAP process causes the formation and separation of subgrains due to the accumulation of plastic strain [33]. Thus, the deformability of ductile metals decreases with increasing the number of passes in the ECAP process [46]. However, the type of bonding at the interface is not affected by the number of ECAP passes [47].

Due to the heterogeneous behavior of the samples, the mean total tensile elongation was burdened with a large error (6.2%). As in the previous samples, the tensile strength was decreased by 15.18 MPa compared to the base material. The yield strength of the ECAP14 sample also decreased by 6.92 MPa.

**Figure 16:**
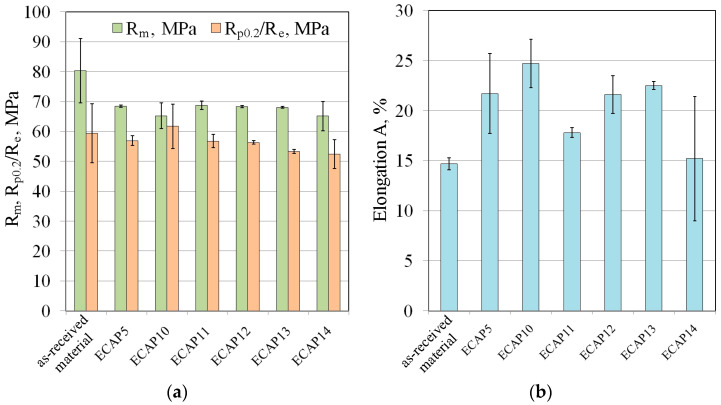
The influence of the type of ECAP process on (**a**) the tensile strength and yield strength, and (**b**) elongation of material.

Figure 16 presents a comparison of the static tensile test results for the base material samples and the ECAP-treated samples. It was observed that both the tensile strength and the yield strength of the material decreased in the ECAP-processed samples (Figure 16a). With each successive pass in the same bimetallic sample orientation (samples: ECAP10, ECAP13, ECAP14), a gradual decrease in the values of yield strength was observed. This indicates a correlation between the number of ECAP passes and the material properties resulting from the influence of work hardening. Analysis of tensile diagrams of Al/Cu bimetallic rods in ECAP presented by Volotikina et al. [48] showed that work hardening leads to an increase in strength properties and a decrease in the elongation of workpieces.

**Figure 17:**
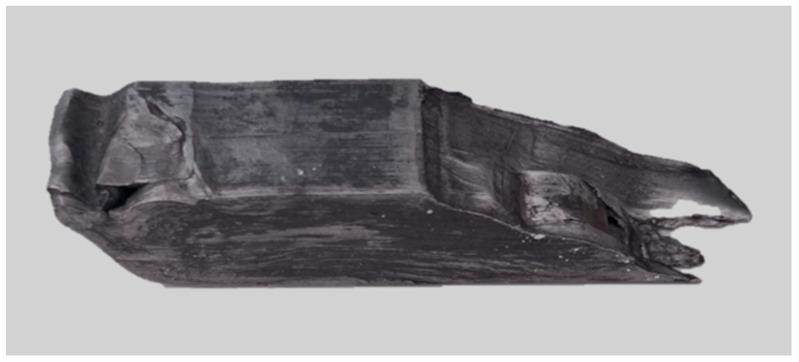
Photograph of ECAP14 sample.

After ECAP processing, the change in the total elongation of the material was also observed (Figure 16b). The average value of this parameter increased after the ECAP processes compared to the explosively welded bimetallic material. The highest elongation of the material among the samples subjected to the ECAP process was recorded for the ECAP10 sample (A = 24.7%). The lowest average elongation value was obtained for the ECAP14 sample (A = 15.2%). Due to the three-pass processing and the associated severe plastic deformation in the material volume (Figure 17), high standard deviations (SDs) were observed for the ECAP14 sample, both for tensile strength, yield strength (Figure 16a), and elongation (Figure 16b).

**Figure 18:**
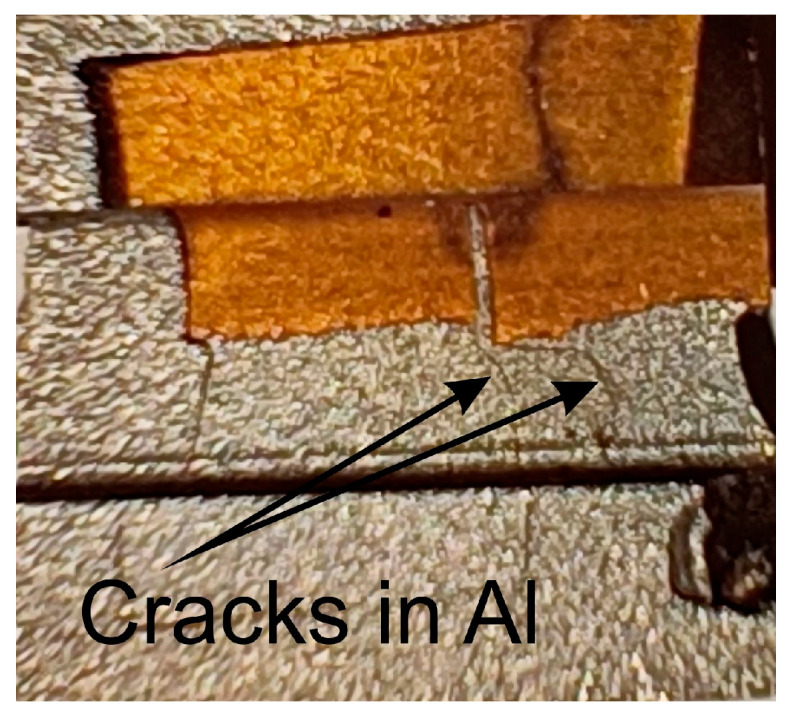
View of the sample in the area of observed macroscopic cracks.

**Figure 19:**
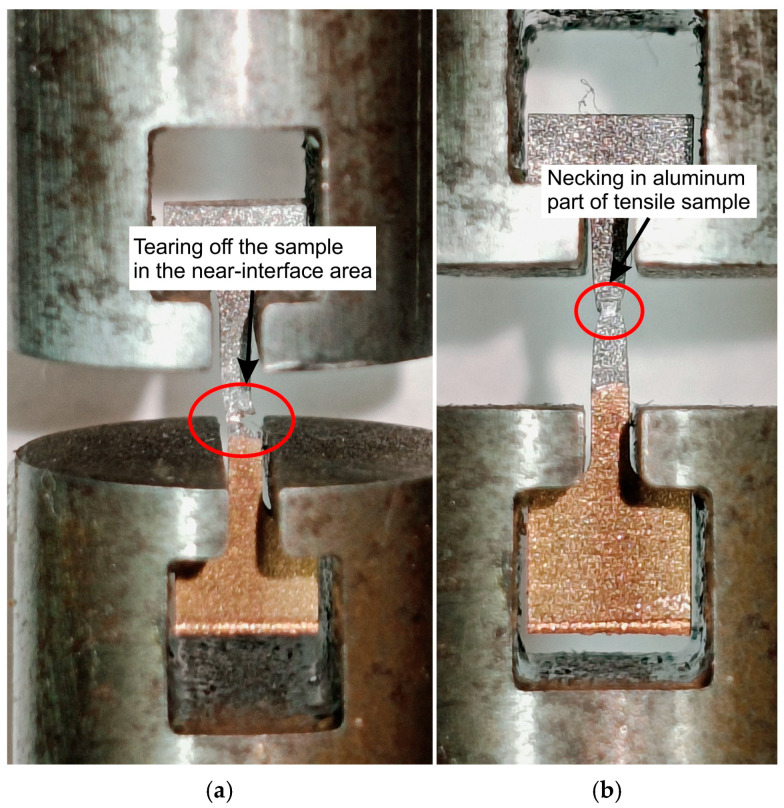
View of the samples after the ECAP process during the tensile test for (**a**) ECAP14 and (**b**) the remaining samples.

The reason for the large standard deviation in the case of the elongation of ECAP14 (Figure 16) was related to the cutting location of the samples for the static tensile test. During the cutting of the samples for tensile tests, cracks, more or less visible to the naked eye (Figure 18), appeared in the material, which were not observed during the cutting of the sample for structural analysis. The samples were cut to avoid these cracks. However, in some tensile samples, invisible micro-cracks may have occurred in the contact line area, which consequently significantly affected the obtained results. Thus, in some of the ECAP14 samples subjected to the tensile test, unlike the others (Figure 19a), cracks occurred near the interface area (Figure 19b). This means that after the third pass using the ECAP method, the deformed bimetallic material began to crack in some places in the joint area, which simultaneously resulted in a decrease in properties compared to the sample after the second pass, and a large scatter in the obtained mechanical parameter values, particularly elongation—as clearly visible in Figure 15e.

Unlike copper, aluminum belongs to the group of materials with high stacking fault energy (SFE), equal to 150–230 mJ/m^2^. This means that it has a high tendency to recover, due to the presence of full dislocations that can easily climb or cross-slip. Consequently, in a strongly deformed material with high SFE, the recovery process may appear earlier, even during plastic deformation [49]. In the case of copper, its SFE is lower, approximately 80 mJ/m^2^, which means that the material has a higher tendency to recrystallize and plastic deformation through twinning. Moreover, twin boundaries are quite stable during recovery. Recovery itself in copper may also be delayed and slower, compared to aluminum, due to the need to apply significant thermal energy for partial dislocations to constrict into full dislocations. In the case of the analyzed bimetallic material, composed of metals with two significantly different stacking fault energies, changes in their structure (grains and subgrains) may be significantly different. In the case of aluminum, which served as a flyer plate here, plastic deformation after explosion welding (EW) could have been large enough to cause the appearance of a large enough number of dislocations in the structure that renewal processes could have occurred during the ECAP process. Moreover, in the case of aluminum, the deformation process at room temperature can be treated as warm forming (T/Tm = 0.49, while for copper T/Tm = 0.22), which could have further intensified dislocation movement and the recovery process during extrusion. As a result, the hardness of the aluminum layer could decrease. In the case of the copper layer, the cumulative plastic strain (EW + ECAP) contributed to further material hardening, which may be related to the deformation mechanism for FCC metals with low SFE and the need to apply high thermal energy to initiate recovery processes. When analyzing the mechanical properties of bimetallic samples, attention should also be paid to the location of the neck. In the case of the tested samples (except for ECAP14), the deformation occurred in the center of the aluminum layer—in the area of lowest hardness. Therefore, the mechanical properties of the material determined in this manner depended to a greater extent on the properties of the aluminum layer than on the copper layer or the interface.

#### 3.2.2. Vickers Hardness After ECAP

**Figure 20:**
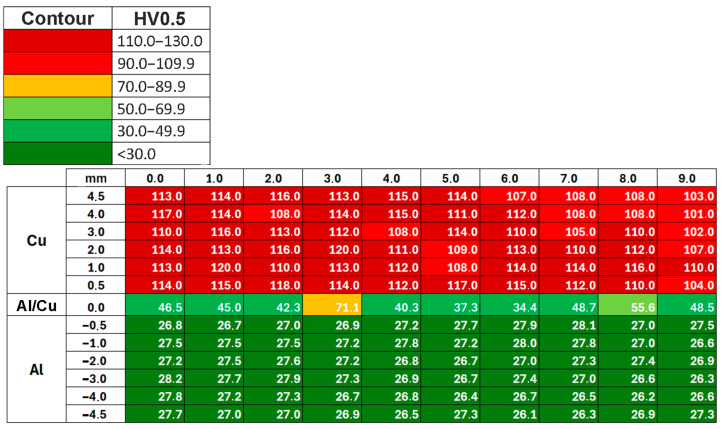
Vickers hardness distribution map for the ECAP5 sample (Al/Cu—the Al/Cu interfacial transition layer).

**Figure A1:**
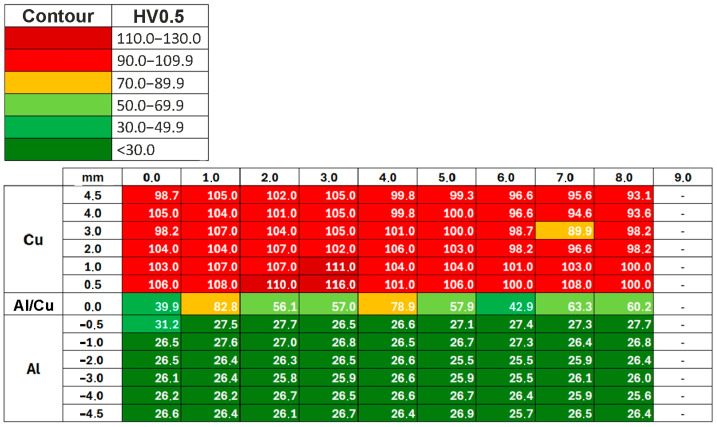
Vickers HV0.05 hardness distribution in the ECAP10 sample (Al/Cu—the Al/Cu interfacial transition layer).

**Figure A2:**
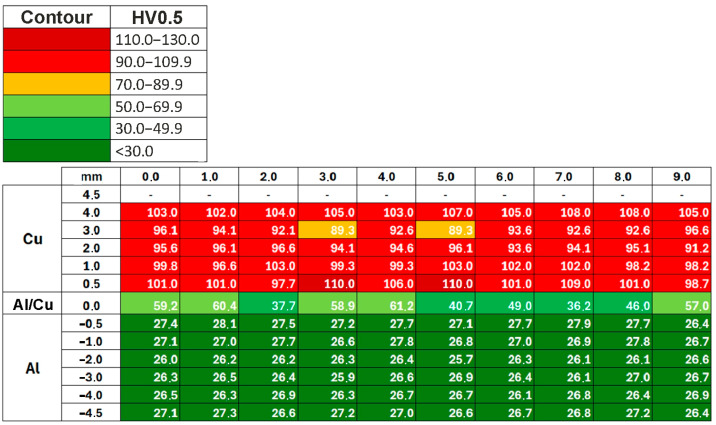
Vickers HV0.05 hardness distribution in the ECAP11 sample (Al/Cu—the Al/Cu interfacial transition layer).

**Figure A3:**
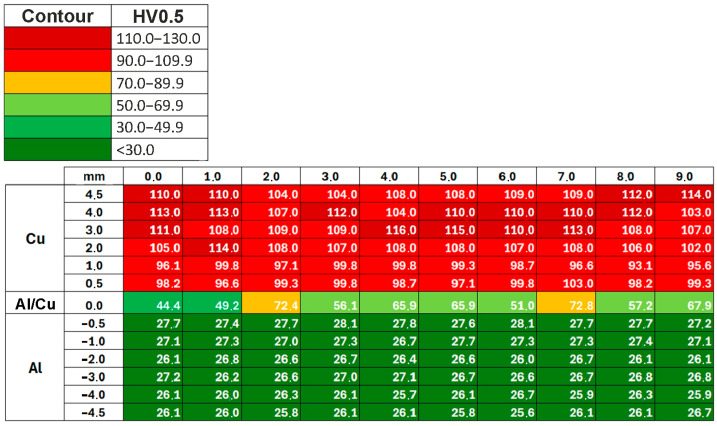
Vickers HV0.05 hardness distribution in the ECAP12 sample (Al/Cu—the Al/Cu interfacial transition layer).

**Figure A4:**
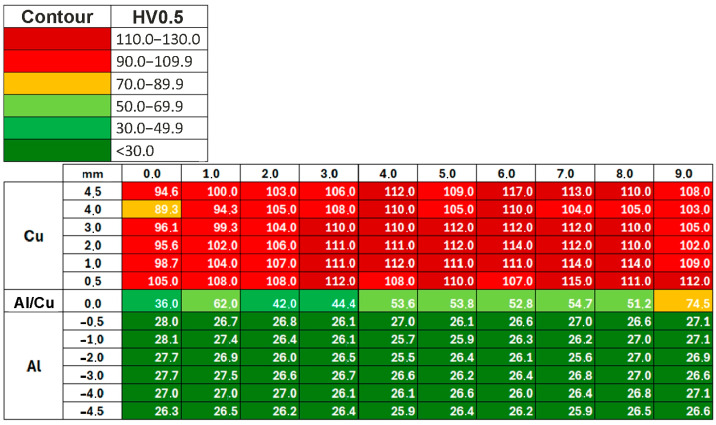
Vickers HV0.05 hardness distribution in the ECAP13 sample (Al/Cu—the Al/Cu interfacial transition layer).

**Figure A5:**
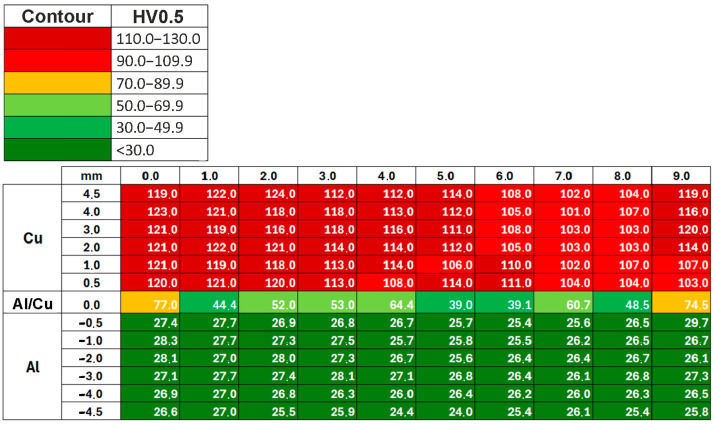
Vickers HV0.05 hardness distribution in the ECAP14 sample (Al/Cu—the Al/Cu interfacial transition layer).

This section presents the results of Vickers hardness measurements of ECAP-processed samples. Hardness distribution maps were analyzed for the Al and Cu layers, as well as for the Al/Cu interfacial transition layer, depending on the sample orientation in ECAP. A color scale was created to visualize the hardness gradient on the sample surface. Figure 20 shows the Vickers hardness distribution map for the ECAP5 sample. It was found that, compared to the base material, the hardness achieved more uniform values throughout the entire thickness of the Al and Cu plates. Vickers hardness distribution maps for the remaining samples are presented in Appendix A (Figure A1, Figure A2, Figure A3, Figure A4 and Figure A5).

**Figure 21:**
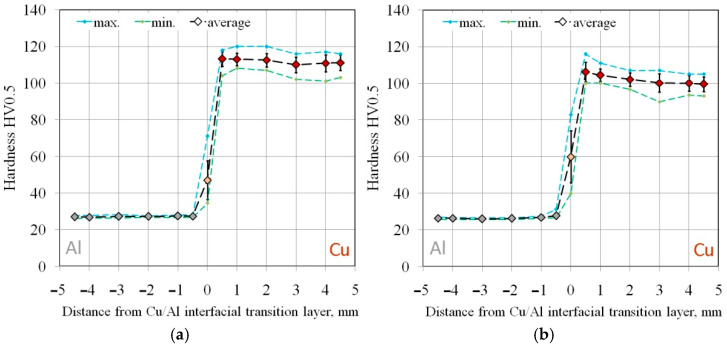

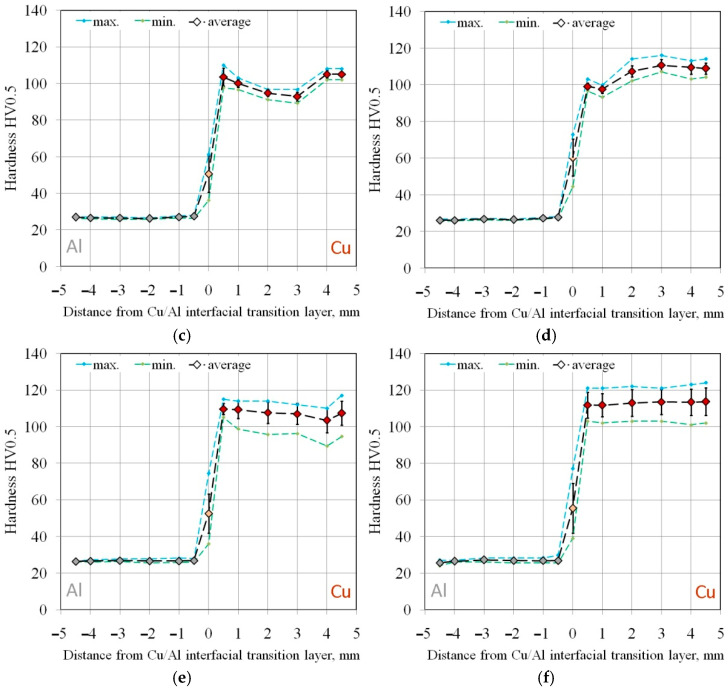
Variation of material hardness depending on the distance from Al/Cu interfacial transition layer for the following samples: (**a**) ECAP5, (**b**) ECAP10, (**c**) ECAP11, (**d**) ECAP12, (**e**) ECAP13 and (**f**) ECAP14.

The average hardness of the ECAP5 sample (Figure 21a) was as follows: Cu layer—111.8 HV0.5 (SD = 4.1 HV0.5), Al layer—27.1 HV0.5 (σ = 0.5 HV0.5), and the Al/Cu interfacial transition layer—47.0 HV0.5 (σ = 10.5 HV0.5). This means that after ECAP processing, the Cu layer hardness increased by 12.9 HV0.5, while the hardness of the Al layer and the Al/Cu interfacial transition layer decreased by 5.3 HV0.5 and 10.84 HV0.5, respectively, compared to the base material. The average hardness of the ECAP10 sample (Figure 21b) was as follows: Cu layer—102.1 HV0.5 (σ = 4.8 HV0.5), Al layer—26.6 HV0.5 (σ = 0.8 HV0.5), and the Al/Cu interfacial transition layer—59.9 HV0.5 (σ = 14.2 HV0.5). After ECAP, the hardness in the Cu layer and the Al/Cu interfacial transition layer increased by 1.5 HV0.5 and 3.8 HV0.5, respectively. While the hardness of the Al layer decreased by 5.9 HV0.5 compared to the base material cut longitudinally to the Al/Cu transition layer. After processing the ECAP11 (Figure 21c) sample, the hardness decreased in all layers by 1.3 HV0.5 (Cu layer), 6.2 HV0.5 (Al layer) and 5.5 HV0.5 (Al/Cu interfacial transition layer) compared to the base material.

The hardness change for the ECAP11 sample has a different profile in the Cu layer compared to the ECAP5 and ECAP10 samples. In the Al layer, the curve was almost rectilinear, while in the Cu layer, a decrease in hardness was observed in the central part of this layer. This shape resembles the hardness distribution in the base material (Figure 12), where the highest Cu layer hardness values occurred near the interface and then decreased with distance from the Al/Cu interfacial transition layer. However, in the ECAP11 sample, the Cu layer hardness in the area furthest from the Al/Cu interfacial transition layer was comparable to the hardness in its vicinity. The varied hardness distribution in the individual layers of the tested material resulted from the character of the material deformation in the die entrance channel. The ECAP11 sample was processed with the Al plate side oriented towards the channel outlet (Figure 6c). Similar results were obtained by Kaya [42], where the hardness value decreased with distance from the interface and then the hardness value increased again at distances closer to the external surface.

In the case of the ECAP12 sample which was processed with the Cu plate side oriented towards the channel outlet (Figure 6d), the hardness reached the highest values near the outer layers of the material (Figure 21d). The orientation of the samples at the beginning of the ECAP test therefore influenced the hardness distribution in the cross-section of the ECAP-processed samples. The hardness of the Cu layer material and the Al/Cu interfacial transition layer material increased by 4.9 HV0.5 and 4.2 HV0.5, respectively. The hardness of the Al plate decreased by 6.3 HV0.5 compared to the base material.

The hardness of the Al layer material in the ECAP13 (Figure 21e) sample processed in two passes did not change compared to the measurements in the same layer in the ECAP10 sample, which was subjected to only one ECAP pass. On the Cu plate side, a significant increase in hardness was observed, particularly in the central part of the sample. The average hardnesses in the Cu layer material, Al layer material and the Al/Cu interfacial transition layer were 107.3 HV0.5 (σ = 5.7 HV0.5), 26.6 HV0.5 (σ = 0.6 HV0.5) and 52.5 HV0.5 (σ = 10.7 HV0.5), respectively. The hardness of the Cu plate material and the Al/Cu interfacial transition layer increased by 6.8 HV0.5 and 4.2 HV0.5, respectively, while the hardness of the Al layer material decreased by 6.4 HV0.5 compared to the hardness of the base material.

For the ECAP14 sample (Figure 21f), the hardness of the Al plate material reached more uniform values throughout the entire layer thickness compared to the base material (Figure 12). No significant differences in hardness were observed compared to the ECAP10 and ECAP13 samples, which differed from the ECAP14 sample in that they had fewer passes. The hardness of the Cu plate material significantly increased compared to the other samples subjected to the ECAP processing. The average hardnesses of the Cu layer material, Al layer material and the Al/Cu interfacial transition layer were 112.8 HV0.5 (σ = 6.8 HV0.5), 26.6 HV0.5 (σ = 1.0 HV0.5) and 55.3 HV0.5 (σ = 13.6 HV0.5), respectively. After three passes, the hardness of the Cu plate material increased most significantly in all tested samples, increasing by as much as 12.3 HV0.5 compared to the base material. The average hardness of the Al plate material decreased by 6.4 HV0.5, while a slight decrease of 0.9 HV0.5 was found in the Al/Cu interfacial transition layer.

**Figure 22:**
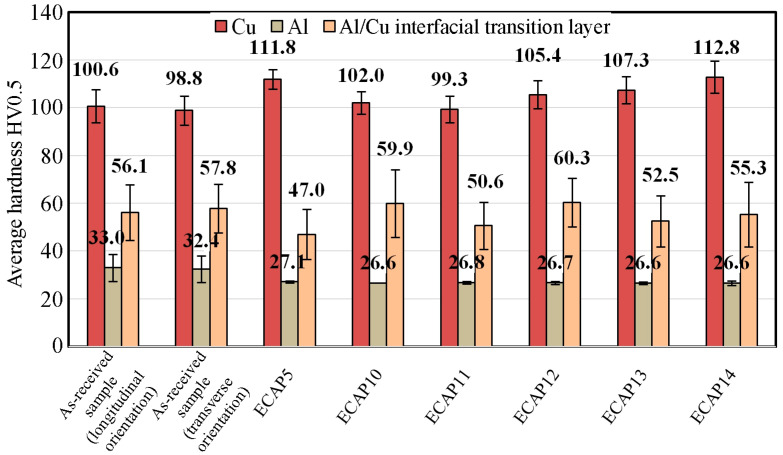
Comparison of the average hardness of ECAP-processed samples with the sample in as-received state.

Figure 22 summarizes the hardness measurement results for all ECAP-processed samples. Data analysis showed that in most cases, the Cu plate hardness increased as a result of plastic deformation. Only the ECAP11 sample showed a decrease in hardness value in the Cu layer compared to the base material (longitudinal orientation), and this difference was 1.3 HV0.5. The hardness in the Al/Cu interfacial transition layer was characterized by the greatest scatter, so large standard deviations had to be considered when analyzing the obtained results. However, only the ECAP5 sample had the lowest hardness at the Al/Cu interfacial transition layer. The most interesting phenomenon was observed in the zone of the Al plate. Regardless of the sample orientation, the number of passes, the hardness values of all samples subjected to ECAP processing were very similar. A slightly higher hardness was observed in the ECAP5 sample compared to the other samples, but these differences were too small to determine the most influential factor. However, a clear effect of severe plastic deformation was observed compared to the base material: regardless of the sample orientation, the hardness of the Al plate material decreased after ECAP processing.

#### 3.2.3. Microstructure After ECAP

**Figure 23:**
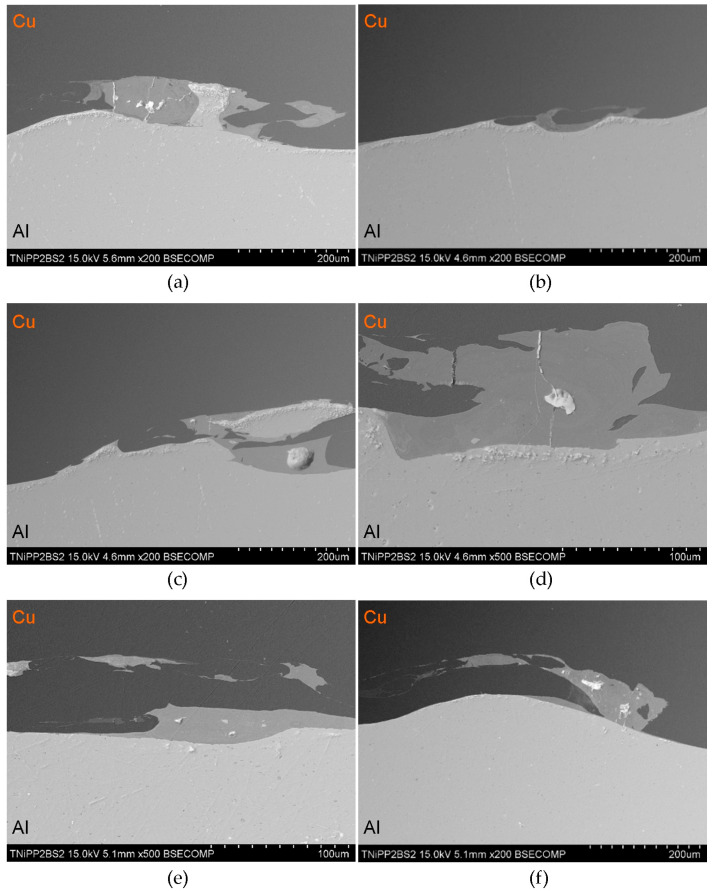
SEM micrographs of the Al/Cu interfacial transition layer of ECAP-processed samples: (**a**) ECAP5, (**b**) ECAP10, (**c**) ECAP11, (**d**) ECAP12, (**e**) ECAP13 and (**f**) ECAP14.

Scanning electron microscopy observations of all samples revealed a distinct phase boundary between the Cu and Al layers. Due to the observation mode used, in most analyses, the copper layer was presented as darker areas, and the aluminum layer as lighter. Transition areas were observed near the Al/Cu interfacial transition layer of the ECAP5 sample (Figure 23a), resulting from local melting of metals during explosive welding. Transition areas were observed near the Al/Cu interfacial transition layer of ECAP10 (Figure 23b), ECAP11 (Figure 23c), and ECAP12 (Figure 23d) samples.

The ECAP5 and ECAP10 samples differed only in the cut plane (Table 3). Therefore, it can be concluded that this parameter had no significant effect on the microstructure of the Al/Cu interfacial transition layer. Analyses of the Al/Cu interfacial transition layer of samples subjected to two and three passes showed a shift of the transition layer towards the Al plate material (Figure 23e,f).

**Figure 24:**
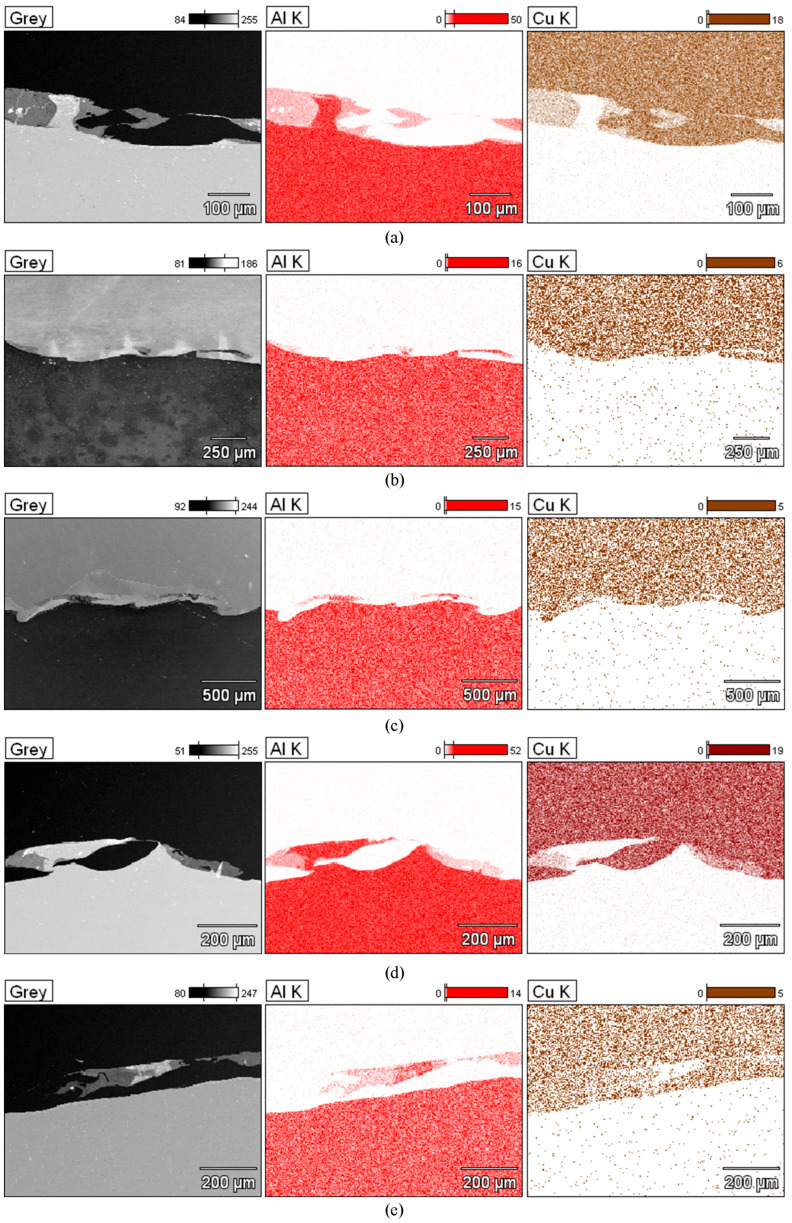
Elementary distribution map in Al/Cu interfacial transition layer: (**a**) ECAP5, (**b**) ECAP11, (**c**) ECAP12, (**d**) ECAP13 and (**e**) ECAP14.

To better qualitatively identify the chemical composition of the phases in the Al/Cu interfacial transition layer, Figure 24 presents the elemental distribution map of ECAP-processed samples. The elemental distribution map in sample ECAP5 (Figure 24a) confirmed a clear interface between the Al and Cu layers. In the central part of the SEM micrograph, an aluminum portion, displaced into the transition zone between the layers, was observed. A clear phase interface, without distinct transition zones, can be observed in the remaining samples ECAP11-ECAP14 (Figure 24b–e). Above the Al/Cu interfacial transition layer in sample ECAP12 (Figure 24c), elongated fragments rich in copper elements were observed in the Cu layer. In samples subjected to multiple ECAP processing, transition zones were detected, representing a mixture of atoms of both base metals (Figure 24d,e).

Table 6 compares the chemical composition of the Al/Cu interfacial transition layer of ECAP-processed samples with the same orientation but differing in the number of passes. In points 1 and 2 of the sample deformed in a single pass, the Cu layer consisted primarily of copper (>99 wt.%) with trace amounts of aluminum (≤0.30 wt.%). Points 3 and 4 were located in the transition region, where the content of Cu (on the Cu plate side) and Al (on the Al plate side) predominated. These are zones of molten material layers in which intermetallic compounds were formed. Point 5 was located inside the Al plate (Al—98.90 wt.%, Al—1.10 wt.%). In the sample deformed in two passes, in points 1 and 2, the material consisted primarily of copper (>99 wt.%) with trace amounts of Al (<0.70 wt.%). Points 3 and 4 are located in the transition region, where the Cu content was slightly predominant. It should be noted that these points were located inside the copper plate. Measurements at points 5–7 showed a clear predominance of Al atoms (>98.00 wt.%) over copper (<2.00 wt.%). At points 1–3 of the Cu plate, in the sample subjected to the triple ECAP process, mainly copper (>99.40 wt.%) with trace amounts of aluminum (<0.60 wt.%) was observed. Point 4, where aluminum (>97.00 wt.%) was detected, was located in the center of the transition region. However, the measurement at point 5, i.e., in the area surrounding the aluminum portion, indicated the presence of elements from both materials being explosively welded. Measurements at points 6 and 7 were taken further away from the Al/Cu interfacial transition layer; therefore, their composition was predominantly aluminum (>98.80 wt.%).

According to the Al-Cu phase diagram and analysis of the chemical composition of the tested joints, several intermetallic phases could have formed [50,51,52]. In [53] the authors indicate that electron diffraction and TEM/EDX chemical composition measurements revealed three crystalline equilibrium phases of the γ-Al_4_Cu_9_, η-AlCu, and Θ-Al_2_Cu types (the latter being dominant). However, most of the observed phases of the general Cu m Al n type (also crystalline) do not appear in the equilibrium Al-Cu phase diagram. Based on the atomic fractions of both elements, it can be concluded that the following phases occur in the Al/Cu system after the explosive welding and ECAP processes: the upper and lower layers (for each tested sample) are regions corresponding to copper and aluminum. Analysis of the local melted area indicates that it consists of both elements simultaneously. Depending on the atomic fraction of these elements, it can be concluded that these are intermetallic phases of the AlCu or Al_2_Cu type. At the same time, the studies showed an increase in the aluminum content in the ECAP13 P3 and ECAP 14, P5 regions (Table 6). According to the Al–Cu phase diagram, the amount of aluminum in these regions corresponds to the chemical composition for the eutectic, the relative amounts of Al/Al_2_Cu.

The results clearly indicate mixing of the bonded materials of the bimetallic plate in the vicinity of the Al/Cu interfacial transition layer. The ECAP process can consolidate particulate metals, causing deformation-induced atom mixing and enhanced diffusion [54]. Severe plastic deformation triggers hardening of the material, which becomes brittle and, as a result of material shearing in the ECAP die, moves in dislocation slip bands [55]. The increased interaction of mobile dislocations induced by shear deformation in the ECAP process has a significant impact on the strain hardening behavior [48].

## 4. Conclusions

This paper presents the results of experimental studies of the mechanical and microstructural properties of explosion-welded Al/Cu bimetallic samples subjected to the ECAP process. Differences between the properties of samples cut transversely and longitudinally to the Al/Cu interfacial transition layer were examined. Based on the conducted investigations, the following conclusions can be drawn:

- Chemical composition
  - Isolated pores and transition regions were present near the Al/Cu interfacial transition layer, resulting from local phase fusion during the welding process. The plane of sample cutting relative to the Al/Cu interfacial transition layer had no effect on the microstructure of the resulting bimetallic plate, which was homogeneous in all three locations of the base material. Sample orientation with respect to the Al/Cu interface had no effect on the microstructure of the base plates.
  - Each subsequent pass in the ECAP process led to a gradual, severe change in the morphology of the Al/Cu interfacial transition layer. As a result of explosive welding, intermetallic compounds were formed only in the Al/Cu interfacial transition layer. As the distance from the Al/Cu interfacial transition layer increased, the foreign phase content in the base material layer decreased.
- Hardness
  - The highest hardness values were obtained in the vicinity of the Al/Cu interfacial transition layer of the base materials. Areas closer to the outer surfaces of the samples had the lowest hardness values.
  - The orientation of the cutting plane of the samples from the base bimetallic material was shown to have no effect on its hardness.
  - Vickers hardness tests of ECAP-processed samples revealed a more uniform hardness distribution compared to the base material. The orientation of the Al/Cu plate layers in the die channel clearly determined the character of the hardness distribution.
  - After ECAP processing, the hardness in the Cu layer increased in all samples as a result of plastic deformation. Regardless of the sample orientation in the die entry and the number of passes, the hardness values in the Al plate of all ECAP-processed samples were very similar and lower than those measured in the base material.
- Tensile properties
  - In its as-received state, the fabricated Al/Cu bimetallic plate was characterized by the highest tensile strength. After the ECAP processing, the tensile strength of all analyzed samples decreased by approximately 15%.
  - During the static tensile test, no material fracture occurred in the Al/Cu interfacial transition layer.

Optimizing the ECAP process on an industrial scale involves ensuring high processing efficiency, microstructural uniformity, and good mechanical properties of components. Key ECAP process parameters include die geometry, processing route, number of passes, ram speed, lubrication conditions, and temperature. A smaller channel angle ensures higher strain per pass and greater grain refinement. The corner angle affects strain gradients and material homogeneity. The processing route determines how the sample is rotated between passes. In general, a processing route with rotation provides a better combination of strength and ductility and a more uniform microstructure. Cumulative strain increases with the number of passes, leading to a finer microstructure and increased strength and hardness. However, a large number of passes leads to significant work hardening of the material and the risk of crack formation. A slower ram speed is beneficial for better microstructural control but reduces processing efficiency. Conversely, high speeds may cause non-uniform deformation. Efficient lubrication reduces the forming force and facilitates material flow. Due to the synergistic effect of multiple parameters on the properties of ECAP-processed materials, the selection of optimal ECAP process parameters should be related to one of the industrial goals: maximizing strength with moderate ductility, maximizing ductility while maintaining good toughness, or maximizing processing speed. All of these goals are subject to additional optimization requirements.

## Figures and Tables

**Table 1:** Chemical composition of Cu-ETP material (wt.%) in accordance with the EN 1652 standard [28].

Bi	O	Pb	Others	Cu
≤0.0005	≤0.04	≤0.005	≤0.03	≥99.90

**Table 2:** Chemical composition of EN AW-1050 material (wt.%) in accordance with the EN 573-1 standard [29].

Zn	Si	Mg	Fe	Ti	Cu	Mn	Others	Al
≤0.07	≤0.25	≤0.05	≤0.40	≤0.05	≤0.05	≤0.05	≤0.03	≥99.50

**Table 3:** Sample designations for ECAP testing.

Sample Denotation	Sample Type	Number of Passes	Sample Orientation at the Entrance to the Die Channel in the First Pass	Processing Method
ECAP5	transverse	1	Al/Cu plate	no sample rotation
ECAP10	longitudinal	1	Al/Cu plate	no sample rotation
ECAP11	longitudinal	1	Al	no sample rotation
ECAP12	longitudinal	1	Cu	no sample rotation
ECAP13	longitudinal	2	Al/Cu plate	180° rotation between successive passes
ECAP14	longitudinal	3	Al/Cu plate	180° rotation between successive passes

**Table 4:** Summary of the results of the hardness measurements of the base material.

Section Orientation	Measurement Number	Average Hardness HV0.5			Standard Deviation HV0.5		
		Cu	Al	Al/Cu Interfacial Transition Layer	Cu	Al	Al/Cu Interfacial Transition Layer
longitudinal	L1	102.8	32.6	53.8	6.7	6.1	12.0
	L2	97.9	32.9	49.0	6.9	5.1	13.9
	L3	101.1	33.5	65.6	7.2	5.6	9.0
	average	100.6	33.0	56.1	6.9	5.6	11.7
transverse	T1	98.6	32.4	65.7	5.4	5.6	14.9
	T2	98.1	32.5	53.3	6.8	5.6	12.4
	T3	99.8	32.3	54.4	6.2	5.2	3.3
	average	98.8	32.4	57.8	6.1	5.5	10.2

**Table 5:** Basic mechanical properties of Al/Cu plate.

**Parameter**	**Sample**			**Average Value**	**Standard** **Deviation**
	**P1**	**P2**	**P3**		
R_p0.2_, MPa	90.5	73.7	73.2	59.4	9.8
R_m_, MPa	92.8	74.7	73.5	80.3	10.8
A, %	15.4	14.3	14.4	14.7	0.6

**Table 6:** Percentage of Al and Cu elements in the samples ECAP10, ECAP13 and ECAP14.

**Sample**	**Point**	**Wt.%**		**At.%**		**Al-Cu** **Phase**
		**Al-K**	**Cu-K**	**Al-K**	**Cu-K**	
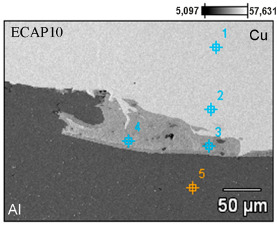	1	0.30	99.70	0.70	99.30	Cu
	2	0.11	99.89	0.25	99.75	Cu
	3	45.34	54.66	66.14	33.86	Al_2_Cu
	4	39.68	60.32	60.77	39.23	Al_2_Cu
	5	98.90	1.10	99.53	0.47	Al
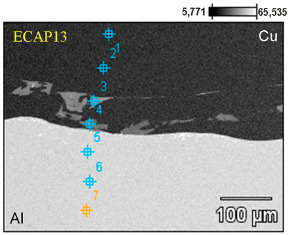	1	0.63	99.37	1.47	98.53	Cu
	2	0.53	99.47	1.24	98.76	Cu
	3	49.81	50.19	70.03	29.97	Al/Al_2_Cu
	4	40.58	59.42	61.67	38.33	AlCu or Al_2_Cu
	5	98.48	1.52	99.35	0.65	Al
	6	98.05	1.95	99.16	0.84	Al
	7	98.81	1.19	99.49	0.51	Al
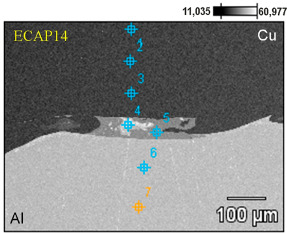	1	0.25	99.75	0.59	99.41	Cu
	2	0.55	99.45	1.28	98.72	Cu
	3	0.25	99.75	0.58	99.42	Cu
	4	97.84	2.16	99.07	0.93	Al
	5	59.10	40.90	77.29	22.71	Al/Al_2_Cu
	6	98.98	1.02	99.57	0.43	Al
	7	98.84	1.16	99.50	0.50	Al

## Data Availability

The original contributions presented in this study are included in the article. Further inquiries can be directed to the corresponding authors.

## Appendix A

Figure A1, Figure A2, Figure A3, Figure A4 and Figure A5 show Vickers hardness HV0.05 distribution in the ECAP10–ECAP14 samples.
